# An Insect-Scale Flapping-Wing Micro Aerial Vehicle Inspired by Tumblers Capable of Uncontrolled Self-Stabilizing Flying

**DOI:** 10.34133/research.0787

**Published:** 2025-07-31

**Authors:** Xiang Lu, Yulie Wu, Jie Chen, Yang Chen, Xuezhong Wu, Dingbang Xiao

**Affiliations:** ^1^ College of Intelligence Science and Technology, National University of Defense Technology, Changsha 410073, China.; ^2^ National Key Laboratory of Equipment State Sensing and Smart Support, National University of Defense Technology, Changsha 410073, China.

## Abstract

As an emerging frontier in biomimetic intelligent microsystems, insect-scale flapping-wing micro aerial vehicles (FWMAVs) demonstrate significant application potential due to their exceptional maneuverability and stealth capabilities. This study proposes a novel mechanical self-stabilization architecture validated through systematic engineering design to address the critical challenge of balancing dynamic instability with payload constraints in stable flight control. By integrating a piezoelectric direct-drive actuator to streamline transmission mechanisms with the optimized V-wing configuration, we developed a V-wing FWMAV prototype weighing 204 mg (wingspan: 68 mm) that demonstrates 41.5% enhanced lift performance and 40% reduction in structural asymmetry errors compared to previous iterations. To overcome the inherent limitations of conventional control methods in payload capacity and response latency, we engineered a cylindrically symmetric damping mechanism. Through the symmetrical aerodynamic design of the top layout, this innovation generates 3-dimensional restoring moments through optimized vortex distribution patterns, achieving isotropic damping effects in the vertical axis. Experimental results reveal that the 241-mg Tumbler FWMAV equipped with this damper exhibits breakthrough stabilization performance: Vertical stabilization duration shows 5- and 20-fold improvements over conventional cross-type dampers and undamped systems, respectively, enabling stable untethered hovering flight exceeding 15 s. The established integrated design paradigm combining structural optimization, aerodynamic enhancement, and passive stabilization control provides a new way to the longstanding technical bottleneck between payload capacity and dynamic stability in insect-scale FWMAVs.

## Introduction

The stability control of flapping-wing micro aerial vehicles (FWMAVs) has remained a critical challenge since their inception, with controllability being an essential prerequisite for practical deployment [1]. While autonomous stable flight represents a fundamental requirement for functional utility, achieving this goal in insect-scale FWMAVs presents extraordinary technical hurdles. Like natural hovering systems (e.g., hummingbirds) and rotary-wing counterparts, FWMAVs exhibit inherent dynamic instability during operation. Without closed-loop feedback control, these vehicles typically experience rapid post-takeoff roll instability, rendering sustained hovering unachievable [2]. Although feedback systems can theoretically stabilize flight, their implementation introduces critical trade-offs: Additional sensors, control circuits, and computational payloads exacerbate the already stringent mass constraints (typically <1 g for insect-scale systems) while introducing latency that compromises real-time responsiveness [3]. Regarding the core challenge faced in stable control flight, the contradiction between dynamic instability and load constraints, the studies of relevant institutions mainly focus on enhancing aircraft performance and optimizing flight control algorithms.

Recent advances in miniaturized actuation systems have enabled notable progress in FWMAV untethered flight. Motor-driven platforms like the DelFly series (33 cm wingspan) demonstrate agile maneuvers including rapid turns and aerobatics [4,5], while gear-reduced designs achieve 12-g FWMAV cable-controlled flight [6,7]. Phan’s morphing-wing FWMAV (18 g) further demonstrates autonomous takeoff and landing capabilities [8,9]. However, such motorized systems remain impractical at true insect scales (<10 cm wingspan, <1 g mass), prompting exploration of alternative actuation strategies. Electromagnetic [10–12], piezoelectric [13–16], and dielectric elastomer actuators (DEAs) [17–19] now enable sub-gram FWMAV implementations: The RoboBee series via piezoelectric actuation improved the lift from 1.3 mN to 3.69 mN [20–23], and the untethered open-loop flight (0.3 s) was achieved using 3 times the solar intensity to provide energy [24], while Pb(In_1/2_Nb_1/2_)O_3_–Pb(Mg_1/3_Nb_2/3_)O_3_–PbTiO_3_ (PIN-PMN-PT) crystal actuators directly connect the wings to generate a maximum lift–weight ratio of 1.61 [25,26], and a short uncontrolled off-line takeoff (<0.5 s) of the robot is achieved based on radio frequency power supply [27] and battery power supply [28]. DEA-driven systems demonstrate particular promise, with Chen’s 155-mg prototype achieving 1.8-mN lift at 1.3 kV [29], later enhanced robotic lift to 6.01 mN at 630 V through DEA multilayer fabrication improvements [30].

Parallel advancements in flight control methodologies have addressed some stabilization challenges. Ma et al. [21] introduced the split-cycle constant period frequency modulation method to generate yaw moments. They used the optical motion capture system to conduct real-time motion capture and feedback, successfully achieving controllable flight and hovering of Robobee [31]. Chirarattananon’s adaptive control framework extended stable flight durations and successfully achieved stable directional flight, stable takeoff, and landing of Robobee [32]. The Bee++ platform [33,34] demonstrated nonlinear attitude control via Lyapunov-based methods, achieving precisely “∞” shaped trajectory tracking. Chukewad et al. [35,36] adopted a more accurate stroke average force and torque model, and applied it to the 150-mg UW RoboFly, which can achieve stable hovering (root mean square error of 2.5 cm). The linear quadratic regulator (LQR) was used for trajectory tracking at speeds up to 25 cm/s [37]. In the motion capture systems, DEA-driven FWMAVs have enabled collision recovery [38] and achieved 1,000 s of hover flight and can also perform complex flight trajectories and complete double-body flips [39]. Bai et al. integrate a nano-quadcopter and a passive telescopic leg [40], overcoming the limitations of the previous jumping mechanism that relied on standing leg drive, and is used in the DEA-driven FWMAV and improving the efficiency and agility [41]. However, the current stability control of insect-scale FWMAVs mainly uses external sensors and motion capture systems to sense and measure the robotic attitude data to achieve stable control [42–44]. The persistent dependence on external sensing infrastructure and the mass of onboard control electronics continue to impede the development of fully untethered, insect-scale FWMAVs.

Complementary to active stabilization strategies, substantial research efforts have been devoted to developing passive mechanical stabilization mechanisms that circumvent complex control architectures. Early implementations focused on macroscopic ornithopters, with Zdunich’s underbody air damper enabling stable suspended flight in 36-cm wingspan systems [45]. Xu et al. [46] proposed a coordinated driving mechanism and employed optimization control based on reinforcement learning to generate and track the robot’s position and direction from takeoff to landing. In the absence of a high platform, the robot is still able to achieve a relatively long movement range by continuously crawling, jumping, and gliding along the horizontal plane. Breugel et al. [47] and Richter and Lipson [48] subsequently demonstrated combined top-bottom damper configurations generating pendulum-like restoring forces, achieving 30-s stabilized hovering when integrated with feedback control. While Dong et al.’s [49] 3-wing rotorcraft demonstrated damper-assisted torque control in larger platforms, these approaches remained inappropriate for insect-scale implementations. Teoh et al. [50] used aerodynamic dampers for the first time in an insect-scale FWMAV by implementing orthogonally oriented aerodynamic surfaces in sub-gram FWMAVs and employing external visual tracking; this architecture achieved brief (1 to 2 s) height stabilization near desired setpoints.

In this work, building upon our previous development of V-wing piezoelectric direct-drive FWMAVs [51], we propose a V-wing integrated molding scheme based on the structure of X-type piezoelectric direct-drive insect-scale FWMAV [51] proposed in our previous work and optimize the robotic structure, and a prototype with a mass of only 204 mg and a wingspan of 68 mm was developed. The lift performance was improved by 41.5% compared with the previous generation, and the structural asymmetry error was reduced by 40%. To simplify the flight control system, we proposed an air damper with a cylindrical symmetry structure, which can form an isotropic damping effect in the height direction, and it is arranged above the robot to form a Tumbler FWMAV, which realizes the self-stable suspension, anti-interference flight, and mechanical passive somersault and go-around of insect-scale FWMAV in the height direction for the first time under completely open-loop operation.

## Results and Discussion

### Design of anti-interference self-stabilizing Tumbler FWMAV

The passive self-stabilizing damper previously used on MAV is usually a cross-shaped structure arranged at the robot’s upper and lower ends. Under uncontrolled conditions, these dampers could maintain stability in the altitude direction for less than 5 s. In contrast, our cylindrical damper, positioned at the upper section of the robot, offers a substantial improvement in self-stabilizing performance. To reduce the damper’s mass and increase manufacturing efficiency, lightweight and high-strength materials are employed, and an integrated molding process is used during manufacturing.

The damper positions the aerodynamic pressure center above the robotic center of mass, which generates a self-righting moment that keeps the robot stable in the vertical direction while moving. We develop a cylindrical symmetric cylindrical damper. Through the symmetrical aerodynamic design of the robotic top layout, the cylindrical damper forms a 3-dimensional restoring moment by optimizing the vortex distribution, which can form an isotropic damping effect in the height direction of the robot and improve the robotic stability effect in the height direction.

**Fig. 1.**
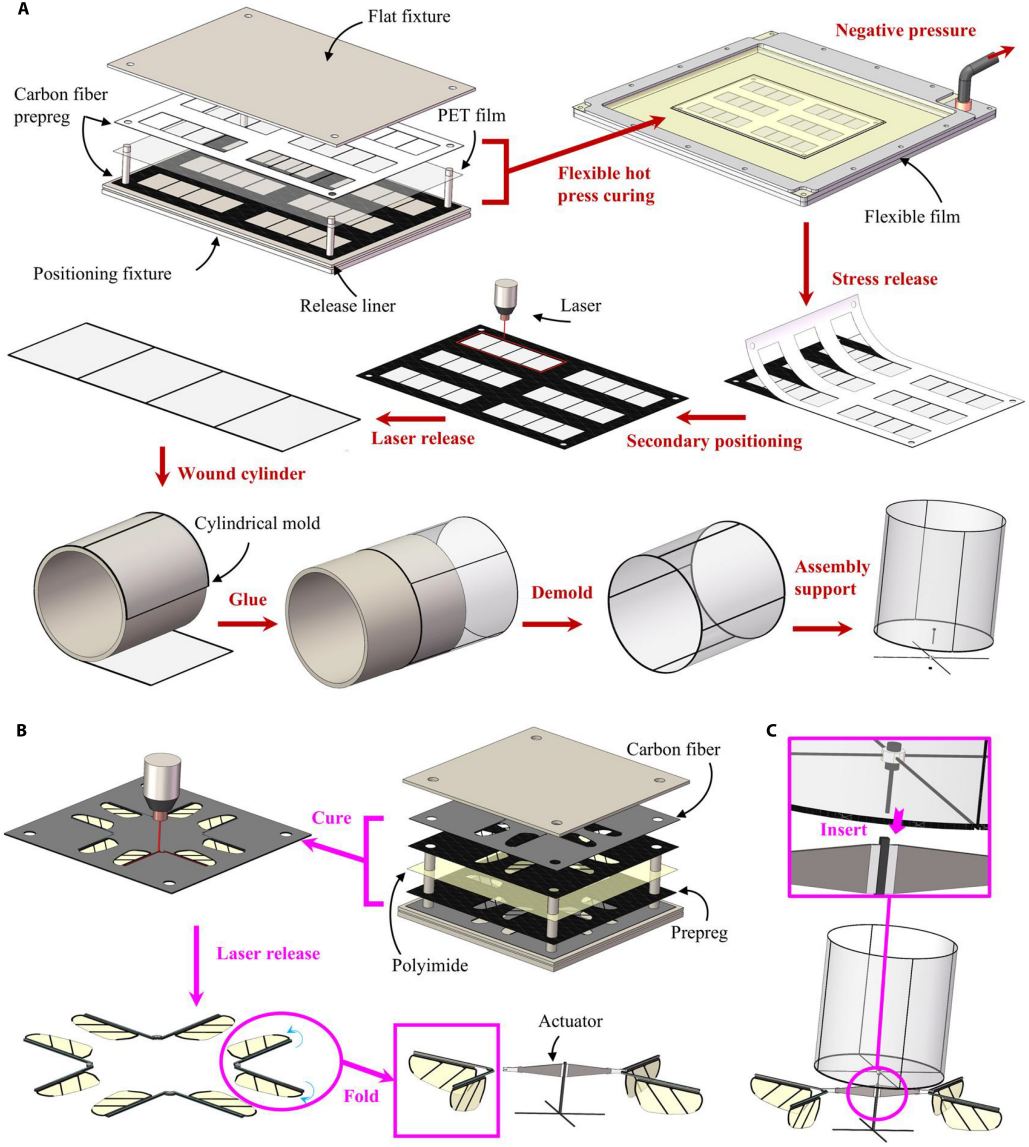
Design and manufacturing of the tumbler FWMAV. (A) Manufacturing and winding process of the cylindrical damper, including laser patterning, composite layer hot pressing curing, structural laser release, cylinder winding fixing, and rotating shaft support assembly. (B) Piezoelectric direct-driven FWMAV V-type passive hinge-wing integrated forming process design. (C) Tumbler FWMAV assembly.

The damper’s cylindrical-symmetric structure is primarily composed of a lightweight, high-strength carbon fiber skeleton and polyethylene terephthalate (PET) film. The supporting components, including carbon fiber rods and resin rings, further enhance its structural integrity. As shown in Fig. 1, the main body of the damper consists of a hollow cylindrical structure surrounded by thin films, which generates self-stabilizing moments through its interaction with the surrounding air. The support assembly includes a cross bracket, a resin bearing, and a rotating shaft. The cross bracket connects the main damper body to the resin bearing, while the rotating shaft links the damper to the robot. This design allows the damper to rotate around the shaft, ensuring a uniform distribution of the self-stabilizing moment.

The Tumbler FWMAV is a piezoelectric direct-driven aerial robot, whose flapping mechanism is based on the X-type piezoelectric actuator design previously proposed by our team [51]. As illustrated in Fig. 1B, the robot primarily consists of piezoelectric actuators, a fuselage, and V-wings. The vibration of the piezoelectric actuators directly drives the V-wings, generating both active flapping and passive pitching motions, which in turn produce the lift force necessary for propulsion.

To assemble the Tumbler FWMAV, the V-wing FWMAV is combined with a damping cylinder. As shown in Fig. 1C, a clamping groove is designed at the top of the robotic fuselage, into which the end of the rotating shaft of the damping cylinder is inserted and secured, completing the Tumbler FWMAV assembly. The final dimensions of the Tumbler FWMAV are 68 mm × 40 mm × 55 mm.

### The static and dynamic characteristics of robot

This section presents the static motion and mechanical characteristics of the V-wing FWMAV. The static motion characteristics primarily involve the active stroke motion and the passive pitch motion of the flapping wings. To measure and analyze the flapping-wing motion, we use a high-speed camera to capture images and data before and after optimization. The driving signal applied is (280 V, 89 Hz, 140 V).

**Fig. 2.**
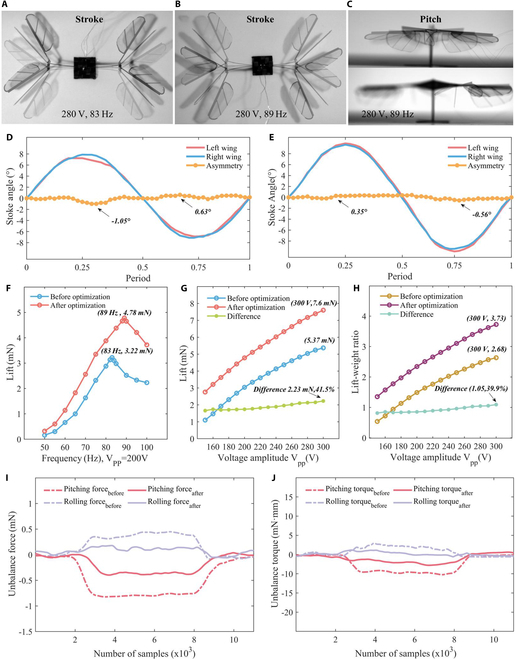
The static and dynamic characteristics of robot. (A and B) High-speed camera view of the robot flapping stroke motion before and after optimization, respectively. (C) High-speed camera view of the robot flapping passive pitch motion. (D and E) Optimization of the robot left and right wing flapping stroke angles of the robot before and after optimization, respectively. The maximum deviation after optimization is reduced from 13.4% to 5.7%. (F) Frequency response comparison curve of lifting force before and after optimization of the robot. (G and H) Drive signal amplitude response curve of robotic lifting force and lift-to-weight ratio at the resonant working frequency. (I and J) Maximum initial rolling force, rolling moment, pitching force, and pitching moment comparison before and after optimization of the robot.

The results, shown in Fig. 2D and E, indicate that the stroke angles of the robotic left and right wings are 9.89° and 9.65°, respectively. Compared to previous studies, the use of a thinner intermediate layer of the actuator and an integrated V-wing design led to a 22.4% increase in the flapping-wing stroke angle. Additionally, the maximum deviation between the stroke angles of the left and right wings is reduced from 13.4% to 5.7%, which can reduce the torque generated by asymmetry during the flapping of the left and right wings. The pitch angle, which is primarily influenced by the width of the passive hinge and the precision of the manufacturing process, remains unchanged, as these parameters are consistent with those used in previous work.

We fix the amplitude of the drive signal (200 V) and conduct a frequency sweep test to identify the robotic optimal operating frequency for flight. As depicted in Fig. 2F, the robotic optimal operating frequency is 89 Hz. A subsequent drive sweep amplitude test, performed at this optimal frequency (89 Hz), revealed that the lift force increases with the amplitude of the drive signal. As shown in Fig. 2G and H, the lift force reached a maximum value of 7.6 mN and the lift–weight ratio reached a maximum value of 3.73 at the highest drive amplitude, representing a 41.5% and 39.9% increase compared to our previous work.

To quantitatively assess the reduction in asymmetry during the flapping motion of the optimized robotic left and right wings, we measure the forces along the *x* axis (pitching force), the *y* axis (rolling force), as well as the torques around the *x* axis (pitching moment) and the *y* axis (rolling moment), under maximum lift drive conditions (300 V, 83 Hz/89 Hz, 150 V). The test results are presented in Fig. 2I and J. At the maximum drive amplitude, the pitching force generated by the optimized robotic structural asymmetry is 0.39 mN, the rolling force is 0.21 mN, the pitching moment is 2.15 mN·mm, and the rolling moment is 1.36 mN·mm. Compared to the robot prior to the improvement, the magnitude of the unbalanced forces and moments caused by structural asymmetry is reduced by more than 40%. These results suggest that structural optimization can improve robotic performance and reduce robotic structural asymmetry errors, which improves the robotic self-stabilizing ability indirectly.

### Uncontrolled hovering flight demonstration

**Fig. 3.**
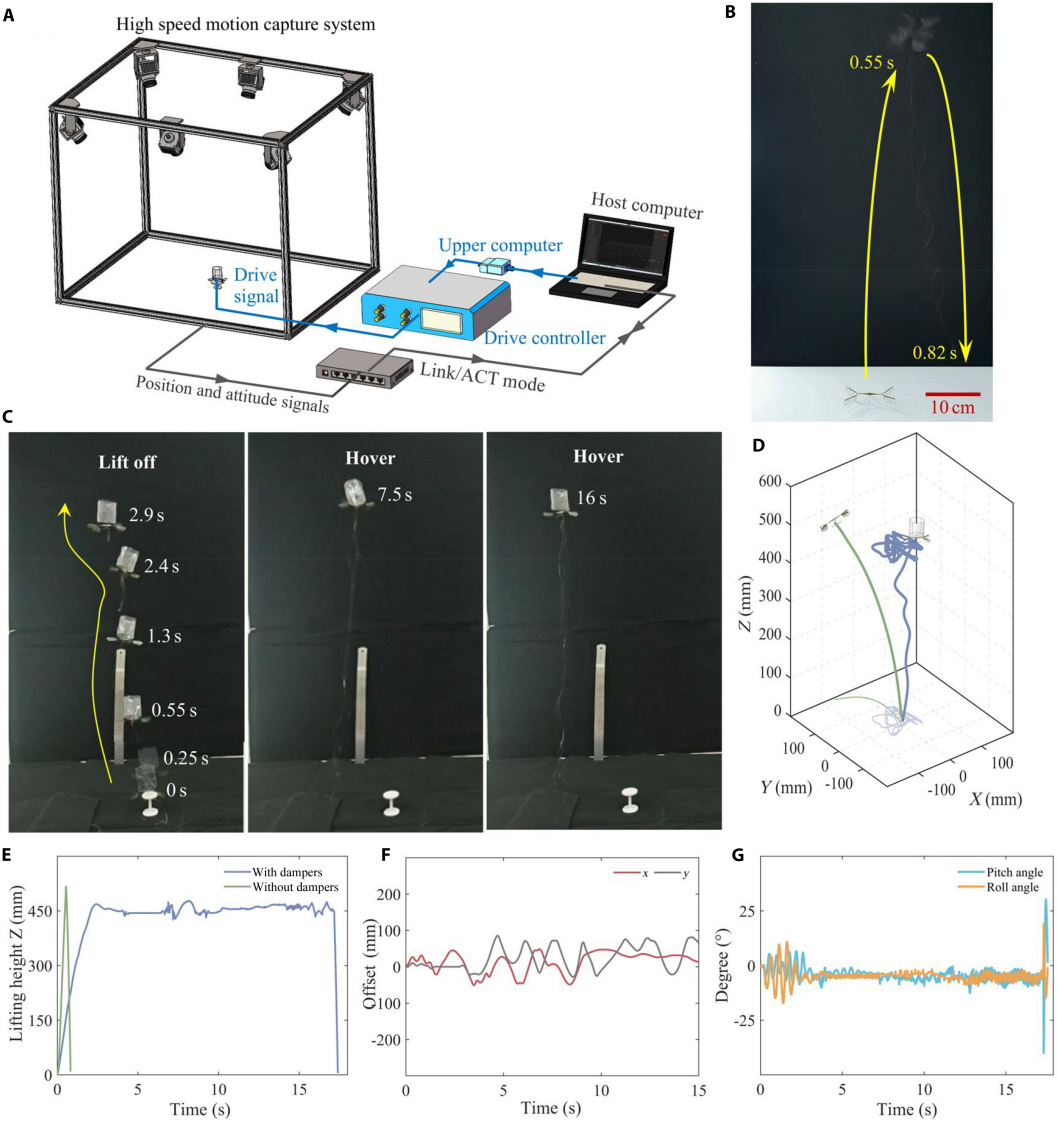
Uncontrolled hovering flight demonstration of Tumbler FWMAV. (A) High-speed motion capture system (working frame rate: 50 fps, capture space: 800 mm × 800 mm × 600 mm). (B) Lift-off demonstration of piezoelectric direct-driven X-shaped FWMAV without damper, which quickly topples after lifting off to the highest point. (C) Lift-off and stable hovering demonstration more than 15 s of Tumbler FWMAV. (D and E) Three-dimensional trajectory fitting and flight tracking altitude–time fitting curve between piezoelectric direct-driven X-shaped FWMAV and Tumbler FWMAV. (F) Position offset tracking fitting of the Tumbler FWMAV in *x* and *y* directions. (G) Pitch and roll angle tracking fitting of the Tumbler FWMAV (all attitude parameters at the robotic takeoff position are set to 0).

To assess the stability improvement of the Tumbler FWMAV in the altitude direction, we conducted an open-loop, uncontrolled stable hovering test. As illustrated in Fig. 3B, a lift-off test was performed on the piezoelectric direct-driven V-wing FWMAV without a damper. The results demonstrated that, under fully open-loop conditions (200 V, 80 Hz, 100 V), the robot experienced roll instability and fell after taking off to the highest point. This instability was attributed to factors such as manufacturing process errors, fuselage coupling torsion, and cable interference.

We conducted a subsequent driving on the Tumbler FWMAV (200 V, 80 Hz, 100 V). As shown in Fig. 3C to E, the robot remained stable in the height direction after takeoff, with a height deviation of less than 38 mm, and the stability was maintained for over 15 s. These results confirm that the cylinder damper importantly enhances the stability of the robot. Regarding horizontal offset motion and attitude angle variations (Fig. 3F and G), the offset distance remained within ±100 mm, and the pitch and roll angle fluctuations were less than 15°.

### Step-up conversion height flight test

**Fig. 4.**
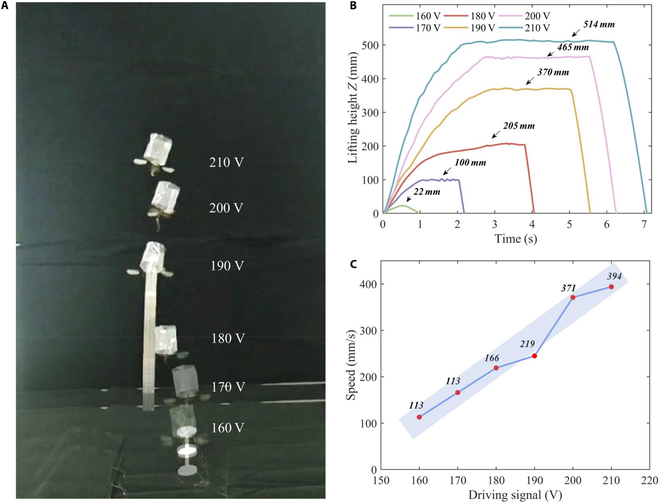
The Tumbler FWMAVs drive signal amplitude-hovering flight demonstration. (A) At the optimal working frequency, robotic flight demonstration under the different drive signal (160 to 210 V). (B) Robot hovering flight altitude tracking under the drive signal. (C) Fitting curve between average takeoff speed and drive signal.

To further validate the repeatability and universality of the Tumbler FWMAV’s stability, we conducted a step-up conversion height flight test. At the robotic resonant driving frequency of 89 Hz, the amplitude of the driving signal (ranging from 160 to 210 V) was gradually adjusted in 10-V increments, altering the robotic lift-to-weight ratio. Figure 4A presents a composite image of the robot during the boosted flight. The lift-to-weight ratios varied with different driving amplitudes, resulting in the robot achieving different hovering heights. As the robot ascended, air resistance and the weight of the cables caused variations in the hover heights at each lift-to-weight ratio. Figure 4B illustrates the relationship between hovering height and driving signal amplitude, while Fig. 4 shows a correlation between the robotic takeoff speed and the driving signal amplitude. The takeoff speed and the driving signal are positively correlated; the fitted relationship is as follows:

$$v_{t}=5.845714\cdot D_{A}-830.1238$$ (1)

where *v_t_* represents the average lift-off speed and *D_A_* denotes the amplitude of the drive signal at the resonant operating frequency.

### Anti-disturbance self-stabilizing flight demonstration

The cylindrical damper experiences resistance perpendicular to the surface when moving horizontally and deflecting, where the Tumbler FWMAVs remain stable in the height direction, have certain anti-interference capabilities, and resume flight in the face of external interference.

**Fig. 5.**
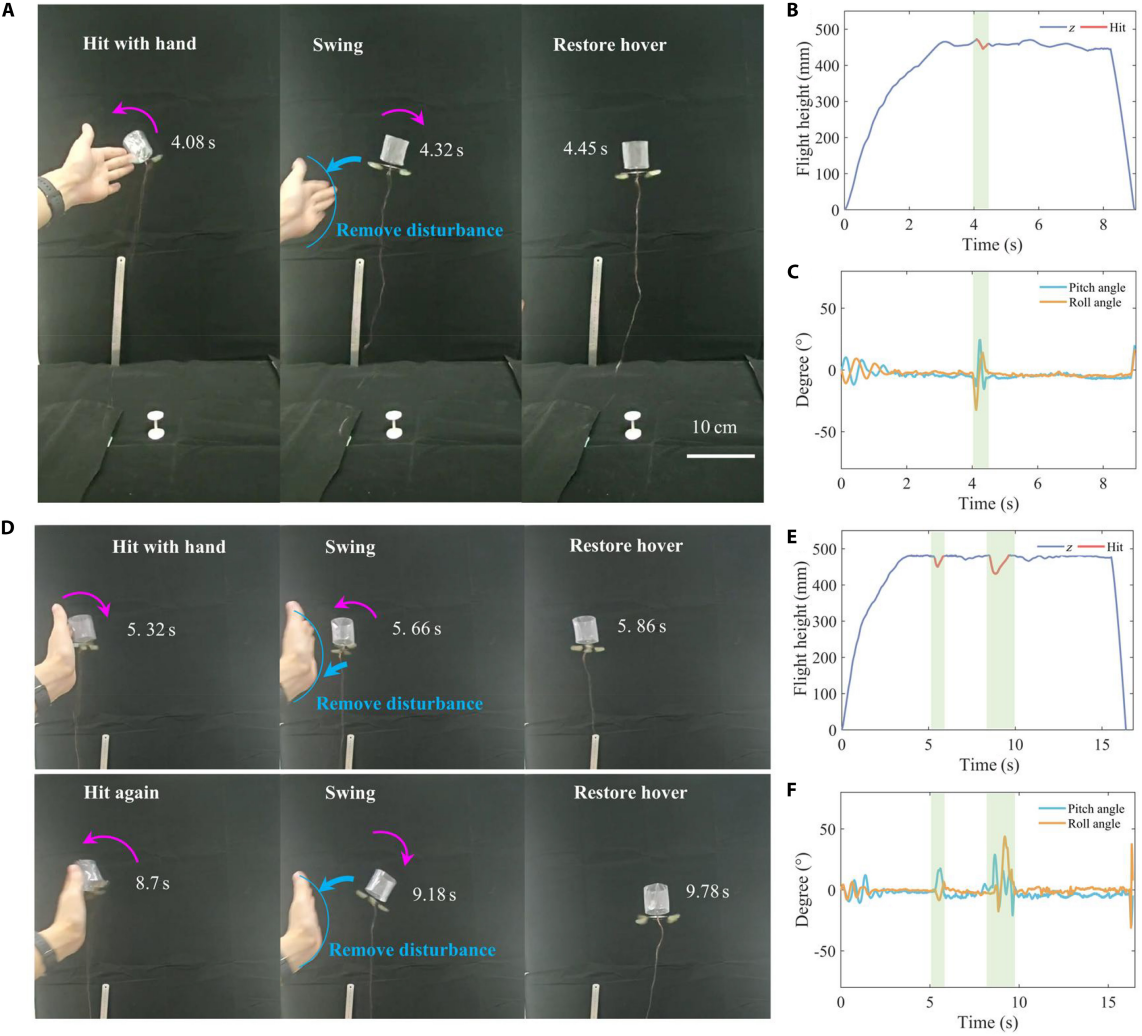
Soft contact impact and collision recovery flight demonstration of the Tumbler FWMAV. (A to C) Flight demonstration and tracking for a single-impact collision recovery. (D to F) Flight demonstration and tracking for twice continuous impact collision recovery. (A) and (D) are flight demonstration diagrams, and (B) is the flight tracking altitude–time fitting curve corresponding to (A). (C) Robot pitch angle and roll angle change corresponding to (A). (E) Flight tracking altitude–time fitting curve corresponding to (D). (F) Robot pitch angle and roll angle change corresponding to (D). The cyan shaded area in the graph represents the collision recovery stage, and the robot resumes its flight attitude within 1 s after soft contact collision interference.

We conducted a collision recovery flight experiment on the Tumbler FWMAV as follows: First, the robot was given a constant driving signal (frame rate: 89 Hz, driving signal: 200 V) to achieve stable hovering for several seconds. Then, we simulated a collision by manually striking the robot, causing it to roll. After the disturbance, the robot resumed stable hovering within 1 s, as shown in Fig. 5A to C. To further validate the robotic performance in recovering stability after a collision, we performed 2 consecutive collision tests on the Tumbler FWMAV. As illustrated in Fig. 5D to F, the robot was hit by a hand after several seconds of stable hovering. Following the first disturbance, the robot recovered from an unstable state back to stable hovering. It was then hit again, resulting in another instability, and the robot returned to the stable hovering flight state within 1 s.

**Fig. 6.**
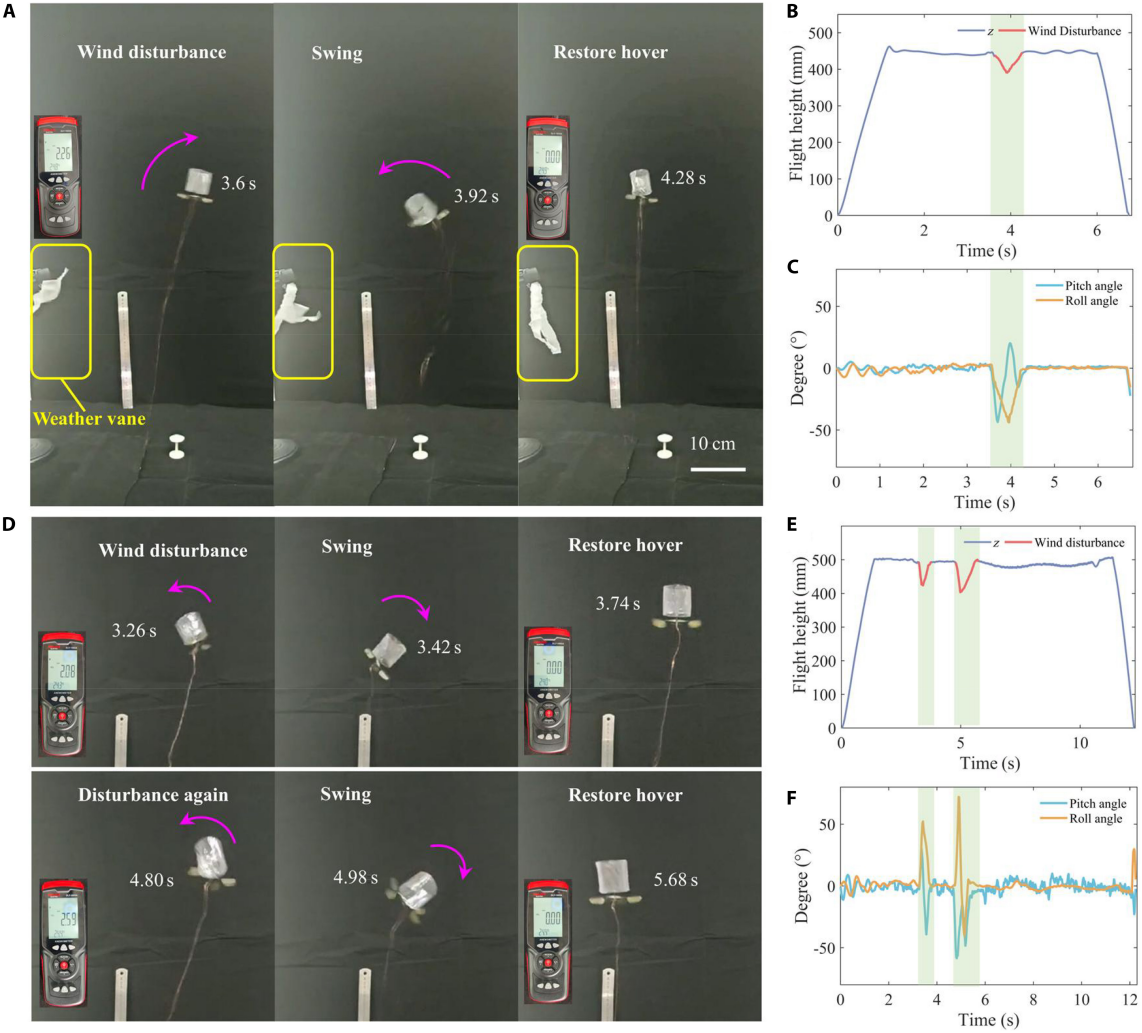
Light gust interference recovery flight demonstration of the Tumbler FWMAV. (A to C) Flight demonstration and tracking for a single light gust (2.26 km/h) interference recovery. (D to F) Flight demonstration and tracking for 2 consecutive light gusts (2.08 and 2.59 km/h) interference recovery. (A and D) Flight demonstration diagrams. (B and C) Flight height tracking and pitch angle and roll angle changes corresponding to (A). (E and F) Flight altitude tracking and pitch angle and roll angle changes corresponding to (D). The cyan shaded area on the graph represents the wind disturbance recovery stage. The robot resumes its flight attitude within 1 s after a wind speed <2.6 km/h gust interference.

Wind resistance disturbance is a critical performance requirement for the development of aerial robots and poses a major challenge for FWMAV applications. The Tumbler FWMAV that we propose demonstrates a degree of resistance to wind disturbances and can return to stable flight conditions after encountering light gusts. Figure 6A shows the robot recovering to stable flight after a gust. The robot was provided with a constant driving signal (frame rate: 89 Hz, driving signal: 200 V), and after several seconds of stable hovering, a fan was used to simulate a gust of wind. The robot quickly recovered from an unstable roll. A wind meter was used to measure the wind speed, which was recorded at 2.26 km/h, and a self-made wind vane displayed the gust conditions in real time, as shown in Fig. 6A. Figure 6B and C depicts the robotic height and attitude changes following a slight gust disturbance. After the wind disturbance, the height fluctuated by more than 55 mm, and the attitude angle fluctuated between −40° and 20°. The robot returned to a stable hovering state within 0.7 s once the gust subsided.

To further verify the wind resistance performance of the Tumbler FWMAV, we conducted 2 consecutive gust interference tests. As shown in Fig. 6D, the wind speeds during the tests were 2.08 and 2.59 km/h, respectively. The robotic height fluctuated by more than 160 mm, and the attitude angle fluctuated between −60° and 75°, as illustrated in Fig. 6E and F. The robot can return to the stable state within 1 s after the fluctuation. These experiments further confirm that the Tumbler FWMAV exhibits strong anti-disturbance self-stabilizing recovery abilities, even under uncontrolled conditions.

### Disturbance somersault and recovery flight demonstration

The V-wing direct-driven structure employed in the Tumbler FWMAV cannot currently generate sufficient rolling torque to induce somersaulting. To assess the robot’s ability to recover its attitude and resume flight after overturning, we introduced external disturbances to provoke a flip.

**Fig. 7.**
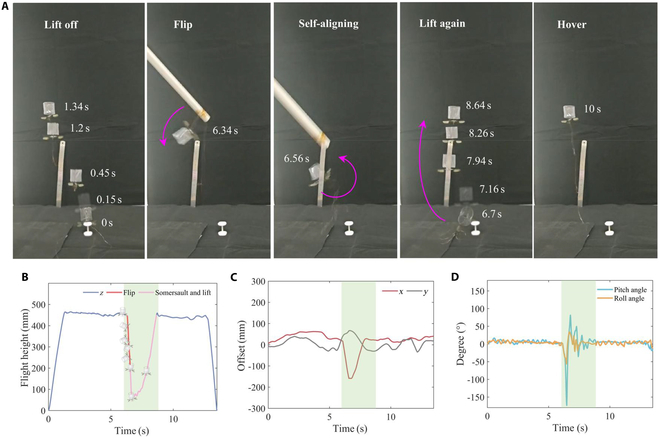
Disturbance somersault and recovery flight demonstration of Tumbler FWMAV. (A) Robotic disturbance somersault and righting flight demonstration diagrams. (B) Flight tracking height–time fitting curve. (C) Position deviation of the robot in *x* and *y* directions. (D) Corresponding change in pitch angle and roll angle of the robot.

Figure 7A illustrates the flight images of the robot following the induced somersault disturbance. The demonstration is divided into 6 key stages: lift-off, stable hovering, disturbance-induced somersault fall, self-correction, ascent, and stable hovering again. The driving signal remains constant throughout the process (operating frame rate: 89 Hz, driving signal: 200 V), with no active control assistance applied. As shown in Fig. 7A and B, the robot promptly lifts off and enters a stable hovering state upon activation of the driving signal. A few seconds later, the robot cables were stirred up through the polyvinylchloride (PVC) pipe, causing it to somersault. Following the flip, the robot falls rapidly due to the alteration in the direction of gravity and lift forces. However, due to the structural characteristics of the Tumbler FWMAV, the robot manages to self-correct and stabilize before reaching the ground. After falling approximately 370 mm, the robot successfully achieves complete attitude self-correction, ascends, and restores its hovering flight. This marks the first demonstration of self-recovery flight in the FWMAV after a mechanically induced passive somersault in an uncontrolled environment, highlighting the Tumbler FWMAV’s good self-stabilization capabilities.

### Longevity performance

The longevity of an FWMAV is primarily constrained by the longevity of the piezoelectric actuator and the fatigue limit of the wings. In the absence of external interference or damage, the fatigue longevity of the wing structure typically exceeds that of the piezoelectric actuator. Consequently, the longevity tests conducted for the robot in this study focus primarily on the piezoelectric actuator and the FWMAV system.

We performed drive tests on individual piezoelectric actuators and FWMAV under the operational conditions (200 V, 80 Hz, 100 V). Tests reveal that the actuator can operate continuously for over 24 h with minimal performance degradation, the operating temperature rises by only 4 °C above ambient conditions, and the FWMAV can remain airborne for more than 40 min without damage.

### Comparison with other advanced research

Remarkable advancements have been made in the research and development of FWMAVs both domestically and internationally, marking major progress in this field. This paper primarily focuses on enhancing the flight capabilities and anti-interference performance of insect-scale FWMAVs. A novel insect-scale FWMAV has been designed and fabricated, featuring passive self-stabilization and anti-interference flight capabilities. The performance of this robot is comparable to the most advanced insect-scale FWMAVs, with its passive self-stabilization and anti-interference flight abilities ranking among the best in the current body of literature.

Numerous motor-driven FWMAVs have achieved cable-free, controllable flight [4–9], with the use of dampers for passive self-stabilized suspension flight [45–48]. However, these robots typically have wingspans exceeding 15 cm and weigh several grams, or even tens of grams. As shown in Table, such vehicles no longer fall within the insect-scale MAV category. In contrast, the latest research on insect-scale FWMAVs has demonstrated cable-controlled flight systems [31–34], with optimal lift-to-weight ratios surpassing 4 [24]. The optimized piezoelectric direct-drive FWMAV developed in this study achieves a maximum lift-to-weight ratio of 3.7, nearing the state-of-the-art in the field. Moreover, the net lift generated by this aircraft reaches 5.5 mN, offering an enhanced load capacity suitable for cable-free deployment. RoboBee is the first insect-scale MAV to utilize double-cross dampers for achieving self-stabilizing suspended flight for durations of 1 to 2 s [50]. In comparison, our proposed cylindrical damper, when integrated with an FWMAV, extends the self-stabilizing suspended flight to over 15 s, nearly an order of magnitude improvement in performance. Additionally, Massachusetts Institute of Technology’s DEA micro-aerial robot has demonstrated a wide range of dynamic maneuvers, including rapid movements under active control, long-duration hovering [39], bouncing [41], somersaults, and recovery post-collision [38]. Building upon cylindrical dampers, our tumbler micro-aerial vehicle is the first insect-scale flapping-wing MAV capable of self-stabilizing after collisions, wind disturbances, and rolling interferences, marking a important progress in the field.

**Table.** Performance comparison of FWMAVs

FWMAVs		Mass (g)	Wingspan (cm)	Lift–weight ratio	The performance of stability
Motor-driven	DelFly Nimble [5]	29.85	33	1.3	No damper; active control; achieving complex movements; no interference immunity test
	Breugel [47]	24	45	1.04	Double cross dampers; hovering more than 30 s; no interference immunity test
	Richter [48]	3.89	14.3	1.16	Double cross dampers and feedback control; hovering more than 80 s; no interference test
Insect-scale	RoboBee series [24,50]	0.070	3	4.1	With double cross dampers can stabilize for 1–2 s; no interference capability
	DEABee [38,39]	0.665	6	3.7	No damper; feedback control, achieving complex movements; the ability to recover after interference
	Bee+ [33,34]	0.095	3.3	1.5	No damper; feedback control; flight stabilized; no interference test
	Ozaki [25–28]	0.120	10.2	1.9	No damper; stable flight was not achieved
This work		0.204	6.8	3.73	Cylindrical damper, hovering more than 15 s, and has certain anti-interference capabilities

## Conclusion

In this paper, we present an uncontrolled self-stabilizing Tumbler FWMAV system based on a novel cylindrical symmetric aerodynamic damper. This design has made breakthrough progress in passive stability mechanisms and flight performance optimization:

· Overcoming limitations of traditional dampers: Insect-scale FWMAVs using traditional cross-type dampers typically suffer from limited altitude stabilization time (<2 s). In contrast, the proposed top-mounted cylindrical damper achieves isotropic force distribution in the horizontal plane, because of its cylindrical symmetry. The damper’s integration of lightweight, high-strength materials and an optimized forming process markedly enhances its self-stabilizing performance in the vertical direction.
· Performance enhancement of FWMAV: Building on the structural optimization of the V-wing piezoelectric direct-driven FWMAV, we developed a 204-mg FWMAV with a 68-mm wingspan. This design improves lift performance by 41.5%, while the structural asymmetry error was reduced by 40%. This progress lays a critical foundation for integrating passive stability systems.
· Integration of the Tumbler FWMAV: We developed a 241-mg Tumbler FWMAV by integrating a cylindrical damper with the optimized FWMAV design. Under constant open-loop driving conditions, the altitude stabilization time of the Tumbler FWMAV exceeds that of the traditional cross-damper configuration by over 5 times, and more than 20 times that of our previous work without dampers. In collision interference tests, the robot recovers to a stable hovering state within 1.3 s and can withstand multiple consecutive disturbances. Wind resistance experiments revealed that, following gust disturbances (wind speed < 2.6 km/h), the system stabilizes within 1 s, markedly improving its environmental robustness.
· Self-recovery flight after mechanical somersault: This research marks the first realization of a self-recovery flight for FWMAVs after a mechanical passive somersault in an uncontrolled state. Through structural dynamics optimization, the robot can correct its attitude after being flipped by external forces, completing rolling, falling, self-correction, and self-stabilizing recovery.

This work introduces a new design paradigm for passive stability mechanisms in FWMAVs. The innovative cylindrical symmetric damper structure lays a strong foundation for developing future micro-autonomous flight systems. While advanced progress has been made, future research must address the following areas:

· Lift performance optimization: The current system has not yet reached its theoretical optimal lift performance. We plan to employ refined fluid–solid coupling modeling and topology optimization algorithms to co-design the material distribution and motion trajectory of the wing membrane. With this approach, we expect a lift-to-weight ratio exceeding 4, providing the necessary load capacity for integrating energy and circuit systems.
· Active control capabilities: The existing system lacks controllable torque generation mechanisms. Future work will focus on developing an aerodynamic/inertia composite control method and establishing a 3-axis active stability control system.
· Untethered flight system implementation: The current wired driving method limits the practical applications of the system. We plan to develop a miniature high-voltage driving circuit based on piezoelectric ceramics, coupled with high-energy-density miniature batteries, to create a complete wireless energy supply solution.

Advancements in the abovementioned research directions will promote the transformation of insect-scale FWMAVs from laboratory prototypes to practical applications, particularly in complex environmental monitoring, disaster rescue, and other critical fields.

## Materials and Methods

### Material choices

#### Wing material

· FWMAV wings comprise rigid wing veins and a flexible wing membrane: The wing skeleton requires lightweight yet high-strength materials, while the membrane must be light, thin, tough, and heat-resistant (withstanding >150 °C). Moreover, wing veins and the membrane should form an integrated composite.​
· Selection and possible alternatives: We selected a 60-μm carbon fiber prepreg (density: 1.73 g/cm^3^) for wing veins. Before curing, it is sticky and soft; after curing, it becomes lightweight and strong. Under hot-pressing, it firmly bonds with the wing membrane, creating an integrated structure. For the wing membrane, 3-μm polyimide (PI) film was chosen. It offers excellent mechanical properties and thermal stability (tensile strength > 200 MPa, usable in −269 to 280 °C). This makes it ideal for the wings’ high-temperature, high-pressure processing and high-frequency vibration environment. As alternatives, <5-μm heat-resistant films like PET can be used. PET films are cheaper but have inferior mechanical properties compared to PI. For wing veins, alternatives include a carbon fiber sheet, polyester glue composite, or glass fiber prepreg, although they are slightly heavier.​

#### Piezoelectric actuator material​

· Piezoelectric actuator materials include ceramic sheets, intermediate adhesive layers, and rigid extension materials: Ceramic sheets should have a piezoelectric coefficient d_31_ <−300 pm/V, tolerate >130 °C, be <0.15 mm thick, and be readily available. The adhesive layer requires strong adhesion, conductivity, high longitudinal rigidity, and tensile strength. Rigid extensions need to be insulating, heat-resistant (>150 °C), lightweight, and strong.​
· Selection and possible alternatives: We chose a 0.13-mm PZT-5H piezoelectric ceramic sheet. With d_31_ = −320 pm/V, dielectric constant 3,800, Curie temperature 225 °C, and density 7.87 g/cm^3^, it meets actuator requirements. The 25-μm unidirectional carbon fiber prepreg serves as the adhesive layer, providing strong bonding, light weight, and high conductivity and tensile strength after curing. The rigid extension is 0.13-mm glass fiber, which is insulating, heat-resistant, lightweight (density: 2.5 g/cm^3^), and strong (elastic modulus > 80 GPa, tensile strength > 2,000 MPa). PMN-PT can replace PZT-5H, with a higher d_31_ (−646 pm/V) but poorer pressure and temperature resistance, fragility, and higher costs. Alumina flakes can substitute glass fiber for rigid extensions, offering higher strength but increased processing difficulty and cost.​

#### Fuselage material​

· Requirements: Lightweight and high strength.
· Selection: We selected a high-modulus carbon fiber composite, which features high strength (elastic modulus > 200 GPa, tensile strength > 3,000 MPa), low density (1.8 g/cm^3^), and low cost (<1 Yuan).​

#### Damper material

· Dampers consist of flexible films and rigid supports: The damping cylinder should be light and strong, the film should be thin, and the support should be strong, tough, bendable, and lightweight, with film–support integration.​
· Selection and possible alternatives: 2-μm PET film was chosen for the damper film. With a density of 1.38 g/cm^3^ and heat resistance of >120 °C, it fits the application. PI films are alternatives (density 1.45 g/cm^3^, heat resistance >150 °C), but 2-μm PI films are costlier than PET.

### Fabrication of robot components

#### Cylindrical damper

Given the strict mass requirements for the robotic system, the damper must be lightweight, as excessive mass would increase the robotic flight load, hindering its ability to achieve independent flight. Therefore, minimizing weight while maintaining high symmetry represents both the focus and challenge in the damper’s design and manufacturing process. To address this, we have developed an integrated processing and winding technique for forming the cylindrical damper, as shown in Fig. 2A. The process includes the following steps:

· Graphical processing of single-layer materials: We utilize an ultra-fast laser (with a processing accuracy of 3 μm and a spot diameter of 10 μm) to pattern the carbon fiber prepreg and PET film.
· Material alignment and curing: The patterned materials are aligned and stacked to form a sandwich structure. These materials are then cured in a flexible coating vacuum chamber, ensuring complete bonding between the prepreg and PET film to form a rigid-flexible composite structure.
· Laser releasing and shaping: Using the patterned positioning holes as guides, we employ the ultra-fast laser to cut and release the composite structure, forming a planar structure of the damper. A cylindrical mold is then used to wind and shape the damper into its final form.
· Assembly: The support structure, consisting of carbon fiber rods and resin bearings, is assembled and fixed through the positioning slots at the bottom end of the damper. This results in the creation of a cylindrical damper capable of rotating around the rotating shaft.

### Piezoelectric actuator

The design and manufacturing process of the piezoelectric actuators were previously discussed in our earlier work [51]. These actuators are composed of PZT-5H piezoelectric ceramic sheets, carbon fiber prepregs, and glass fiber. The carbon fiber prepregs primarily serve for bonding and conduction, with their thickness influencing the output displacement of the actuator. To further enhance the actuator’s displacement, we employed a thinner carbon fiber prepreg (25 μm) as an intermediate structural layer. However, reducing the carbon fiber thickness can weaken the bond between the piezoelectric ceramic sheets and the glass fiber. To counter this, high-temperature-resistant and high-strength acrylic adhesive is applied to the connection after cutting, improving its structural integrity.

### V-wing

We have refined the manufacturing method for the vertical double passive hinge of the V-wing, transitioning from a manual alignment and bonding technique to an integrated folding and fixing process. However, the wings and passive hinge are still manually bonded using adhesive. To further improve the reliability and consistency of the structure, the integration of the V-wing involves forming the hinge and wing as a single structure, which is then folded and fixed. The wings are created by curing a combination of carbon fiber prepreg and PI film. The passive torsion hinge is made by curing 120-μm carbon fiber sheet, a hot-pressed adhesive layer, and PI film. The adhesive layer used for the hinge is the same as the one used to bond the wing’s carbon fiber prepreg cloth, enabling the formation of an integrated structure. Given that the prepreg cloth is thicker than the hot-pressed adhesive layer, the thickness of carbon fiber in the passive hinge is reduced to 100 μm. As shown in Fig. 2B, the improved integration process proceeds as follows:

· Cutting of single-layer materials: Femtosecond lasers are used to cut the shapes and positioning holes of single-layer carbon fiber prepregs, carbon fiber sheets, and PI films.
· Laying and curing multiple layers of materials: A positioning fixture is used to align and stack the cut materials in the order shown in Fig. 2B, followed by curing.
· Cutting and release: The cured layers are released by performing secondary positioning using the pre-made holes and cutting with a femtosecond laser to form a planar structure of the V-wings.
· Folding and fixing: The planar V-wing structure is folded at a 90° angle along the designed hinge and fixed with adhesive to complete the V-wing.

The integrated V-wing is then assembled with the piezoelectric actuator and fuselage to form the V-wing FWMAV, as shown in Fig. 2B.

### Experimental setup for capturing motion characteristics

To facilitate a detailed examination of the robotic flight performance, we utilize a high-speed motion capture system (OptiTrack: NaturalPoint Inc., Corvallis, OR, USA) as depicted in Fig. 6A to obtain complete motion data at high frequencies. The system employs 6 OptiTrack Prime X41 infrared cameras, arranged within a 120 cm × 100 cm × 100 cm capture volume, and supports a maximum frame rate of 1,000 frames per second (fps). Homemade reflective marker balls, each 1.5 mm in diameter, are attached to the robot for position and attitude tracking of the robot. We used a piezoelectric ceramic controller to generate driving signals for the robot, which operates in a fully open-loop control configuration. The driving signals for the 2 actuators of the Tumbler FWMAV are given by:

$$\begin{array}{c} D_{1}\left(t\right)=V_{0}\text{sin}\left(wt\right)+V_{1}\\ D_{2}\left(t\right)=V_{0}\text{sin}\left(wt+\pi \right)+V_{1} \end{array}$$ (2)

where *V*_0_ represents the amplitude of the driving signal, *w* is the frequency of the signal, and *V*_1_ denotes the voltage offset. Both actuators share identical amplitude and frequency values, with only a 180° phase difference between them. By adjusting the amplitude of the driving signals, the robotic lift-to-weight ratio can be controlled.

## Data Availability

The data that support the findings of this study are available from the corresponding author upon reasonable request.

## Supplementary Materials

Supplementary 1 Notes S1 to S7 Figs. S1 to S7 Table S1 Movies S1 to S11
