# Supplementary material for: An Insect-Scale Flapping-Wing Micro Aerial Vehicle Inspired by Tumblers Capable of Uncontrolled Self-Stabilizing Flying
Source: Research (Wash D C). 2025 Jul 31;8:0787. doi: 10.34133/research.0787 (PMC12311305; doi:10.34133/research.0787)
Supplement: Supplementary 1 — Notes S1 to S7 Figs. S1 to S7 Table S1 Movies S1 to S11 [file research.0787.f1.zip › Supplementary meterials.docx]

**Supplementary Materials for：**An insect-scale flapping-wing MAV inspired by tumblers capable of uncontrolled self-stabilizing flying

1Xiang Lu, 1, 2Yulie Wu*, 1Jie Chen, 1Yang Chen, 1, 2Xuezhong Wu, 1, 2Dingbang Xiao*

1 College of Intelligence Science and Technology, National University of Defense Technology, Changsha 410073, China；

2 National Key Laboratory of Equipment State Sensing and Smart Support, National University of Defense Technology, Changsha 410073, China

*Corresponding author. Email: [dingbangxiao@nudt.edu.cn](mailto:dingbangxiao@nudt.edu.cn) .

**The PDF file includes:**

**Notes:**

Notes. S1. Analysis of the robotic aerodynamic self-stabilization mechanism

Notes. S2. Six-axis micro-force measurement system

Notes. S3. Hard contact impact collision recovery flight demonstration of the Tumbler

Notes. S4. Lateral Motion Capability Demonstration

Notes. S5. Verification of Yaw Moment Realization

Notes. S6. The Scalability of the Application and Environmental Adaptability

Notes. S7. Longevity and Maintenance of FWMAV

**Other Supplementary Material for this manuscript includes the following:**

Movies S1 to S11 for multiple supplementary movies：

Movies S1：Flapping wing stroke and pitch motion

Movies S2：Lift-off demonstration without damper

Movies S3：Uncontrolled hovering flight demonstration

Movies S4：Flight under the different drive signal

Movies S5：Soft contact impact and collision

Movies S6：Hard contact impact collision

Movies S7：Light gust interference

Movies S8：Disturbance somersault and recovery

Movies S9：Lateral flight demonstration

Movies S10：Flight demonstration of yaw instability suppression

Movies S11：Hovering without a habitat （cylindrical damper damaged）

**Notes. S1. Analysis of the robotic aerodynamic self-stabilization mechanism**

When the cylindrical damper rolls and deflects, it experiences aerodynamic resistance perpendicular to its surface, resulting in a restoring moment. The aerodynamic resistance is primarily dominated by inertial forces, which are influenced by the damper's structure and the relative flow velocity *v*:

|  |  | (1) |
| --- | --- | --- |

where *ρ* is the air density, is the drag coefficient as a function of the angle of attack , and *A* is the area of the damper.

For small offset speeds, the drag force on both the damper and the robotic wings can be approximated as linearly dependent on the relative velocity of the surrounding air,, and, where *k* is a constant that depends on the aerodynamic damping coefficient and the area of the damper and wings.

To analyze the stability of the Tumbler FWMAV, we employ a first-order approximation method. As shown in **Supplementary FigureS1**, the cylindrical damper is mounted on the top of the robotic system. The installation of the damping cylinder will cause the robotic system to move the center of mass position upward:

|  |  | (2) |
| --- | --- | --- |

is the mass of the damping cylinder, is the total mass of the aircraft system, , and is the mass of the aircraft body.

After installing the cylindrical damper, the distance between the aerodynamic center of each component of the robotic system and the robotic center of mass becomes:

|  |  | (3) |
| --- | --- | --- |
|  |  | (4) |

is the distance from the aerodynamic center of the damping cylinder to the center of mass of the robotic body, and is the distance from the robotic aerodynamic center to the center of mass of the robotic body.

The total moment of inertia of the Tumbler FWMAV is:

|  |  | (5) |
| --- | --- | --- |

where , are the moment of inertia of the robot and the damping cylinder.

Assuming that the robot generates unbalanced disturbances in its hovering state, resulting in roll, pitch(,), and lateral movement(,), aerodynamic forces will produce corresponding drag and moments around the damper and the robot. A dynamic analysis of the system leads to the self-stabilizing control equation:

|  |  | (6) |
| --- | --- | --- |
|  |  | (7) |

where is the acceleration of the lateral movement of the system: , is the lateral force exerted on the damper by aerodynamic forces: , due to the cylindrical symmetry of the damper, the circumferential resistance coefficient *k* remains constant. denotes the lateral force induced by the aerodynamic forces on the robotic wings: , refers to the lateral component of the robotic lift force: , and represent the torques generated by the forces and on the robotic system: , , is the torque generated on the system by the robotic lift force, the lift force passes through the center of mass of the robotic system, so .

Based on the analysis above, the motion control equation for the robotic system in the hovering state () can be derived as follows:

|  |  | (8) |
| --- | --- | --- |

The state equation of the Tumbler robot can be obtained using Equation (8) to derive its motion control equation , that is . Specifically, when the deflection angle of the robotic system in the hovering state is small, the state transition matrix simplifies to:

|  |  | (9) |
| --- | --- | --- |

The characteristic equation of the state transition matrix for the Tumbler robotic system is given by:

|  |  | (10) |
| --- | --- | --- |

According to the design parameters of the robotic system in the table 1, the solutions to the characteristic equations of the system can be solved as [-11.3385 + 0.0000i, -1.4307 + 3.9258i, -1.4307 - 3.9258i] and [-13.8471 + 0.0000i, -2.0164 + 4.2859i, -2.0164 - 4.2859i], all characteristic solutions have negative real parts, and the system is stable in all directions in the horizontal plane, which proved that adding a damping cylinder to the robotic top can ensure the stability of the robotic system in the altitude direction.

**Table S1.** The design parameters of the robotic system

| **Parameters** | **Size** | **Unit** |
| --- | --- | --- |
|  | 38 | mg |
|  | 204 | mg |
|  |  |  |
|  |  |  |
|  | 19.66 | mm |
|  | 11.22 | mm |
|  |  |  |
|  |  |  |

When the Tumbler FWMAV system is hovering, disturbances are applied to alter the initial state of the FWMAV. Two disturbance scenarios are considered: [1 m/s, 1°, 1°/s] and [10 m/s, 10°, 10°/s], simulating the stability response of the robotic system. **Supplementary FigureS1** (b-e) depict the oscillations and subsequent stability recovery process of the robotic system. Due to differences in the robotic moment of inertia around the x and y axes, the recovery times for roll and pitch disturbances vary. Nevertheless, both directions ultimately stabilized.


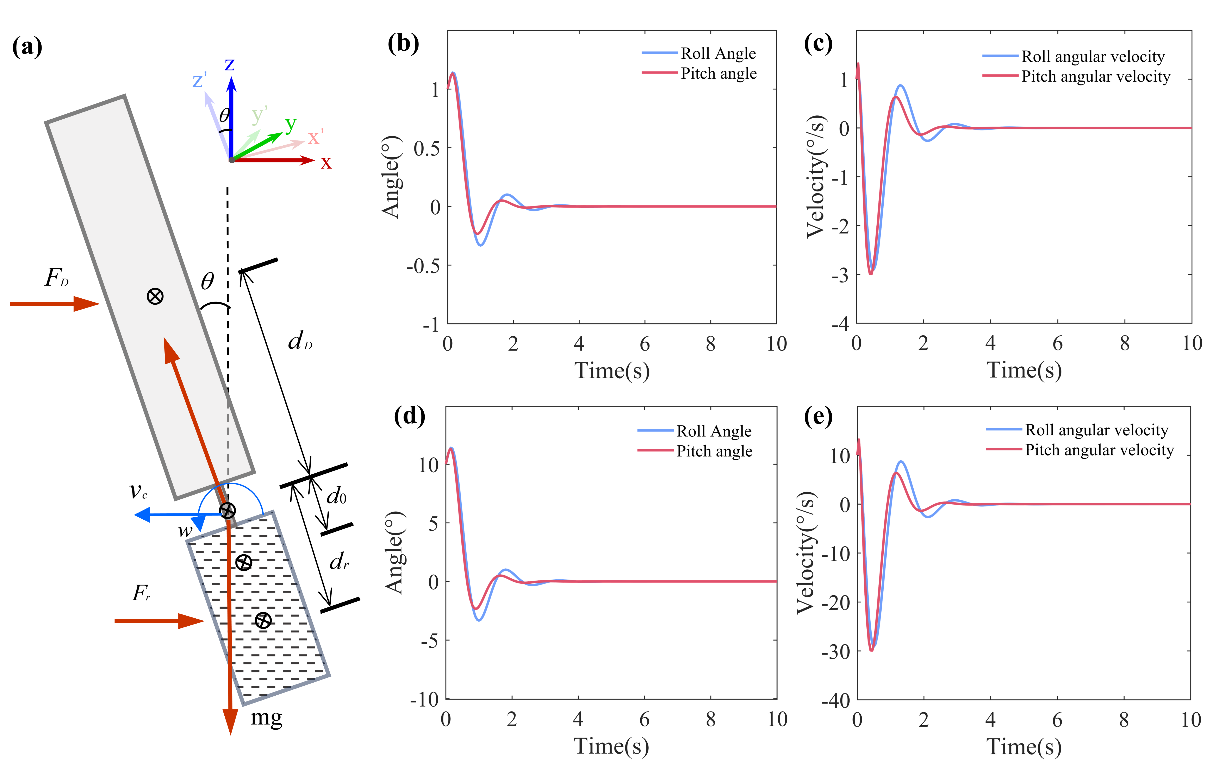


**Supplementary FigureS1:** **Passive self-stabilization analysis of the Tumbler FWMAV:** (a) Model diagram of the lateral force and torque of the Tumbler FWMAV; (b)-(e) The oscillation and stability recovery process of the Tumbler FWMAV around the x-axis (pitch) and y-axis (roll) under different initial external disturbances.

**Notes. S2. Six-axis micro-force measurement system**

The operating frequency of the robot ranges from 70 to 100 Hz, with lift forces on the order of millinewtons (mN). In previous work, we employed a single-axis force sensor to develop a test platform for measuring the robotic lift. However, this device was limited to measuring forces in only one direction and could not capture the asymmetry in forces generated by the left and right wings. For this purpose, we developed a six-axis micro-force measurement device, as illustrated in **Supplementary FigureS2**. This device utilizes a six-axis micro-force sensor as the force-sensitive element, which is mounted on a custom-designed base. The robot is then affixed to the sensor using a specialized fixture, allowing us to measure the forces and moments generated by the robot. The sensor has a response frequency of up to 7 kHz and a resolution of 0.1 mN, which is well above the robotic operating frequency and thus satisfies the testing requirements. We define the robotic coordinate system to fully characterize the dynamic behavior, as shown in **Supplementary FigureS2**. The x-axis is aligned with the actuator's direction, the y-axis is perpendicular to the actuator's face, and the z-axis points vertically upward, along the robotic lift direction.


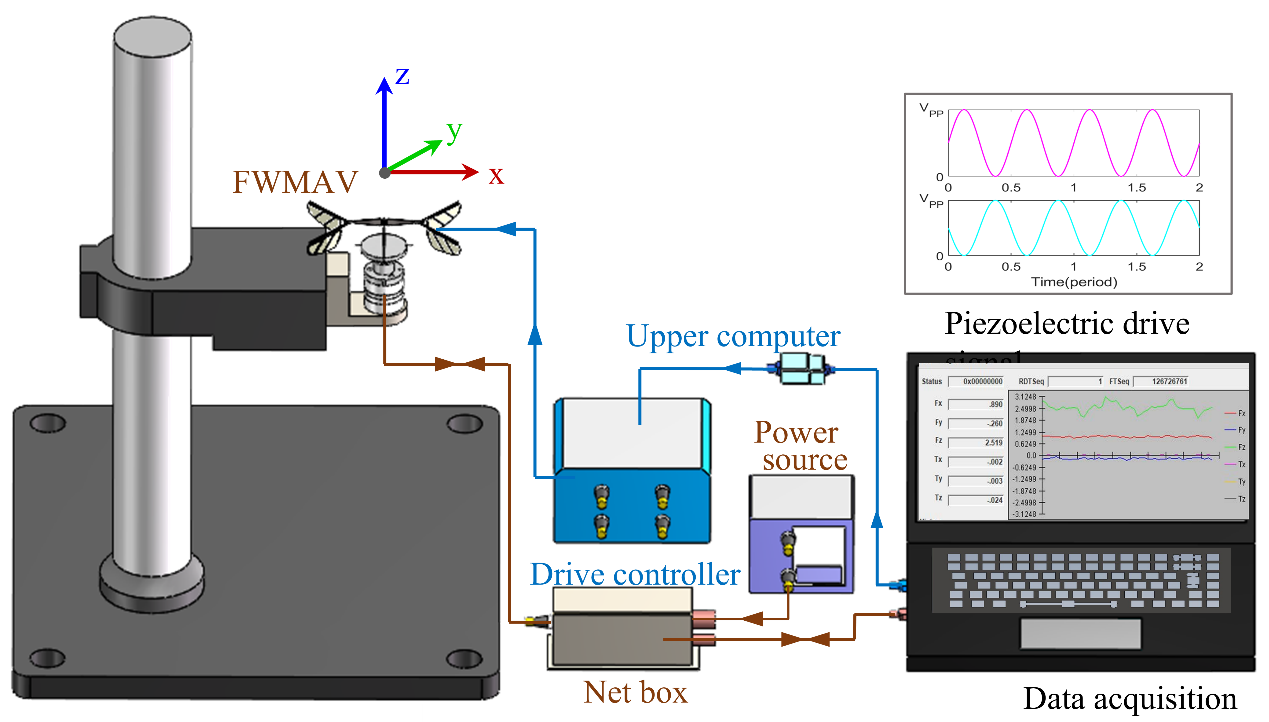


**Supplementary FigureS2:** Six-axis micro-force measurement system (resolution <0.1mN)

**Notes. S3. Hard contact impact collision recovery flight demonstration of the Tumbler FWMAV**

Since the hand is relatively soft, we employed a harder material to simulate collisions with objects of different hardness. Specifically, we used a polyvinyl chloride (PVC) pipe to perform a continuous impact-collision recovery experiment on the robot during hover flight (frame rate: 89 Hz, driving signal: 200V), as shown in **Supplementary FigureS3.(a)**. **Supplementary FigureS3.(b)-(c)** illustrate the fluctuations in the robotic height and attitude after consecutive collisions with the PVC pipe. After each collision, the robotic height fluctuated by more than 70 mm, and the attitude angle fluctuated by more than ±40°. Nevertheless, the robot regained stable hovering flight within 1.3 seconds after each disturbance. All of these experiments were conducted under open-loop, uncontrolled conditions with constant driving parameters. The results demonstrate that the Tumbler FWMAV exhibits robust self-stabilizing recovery capabilities after collisions.


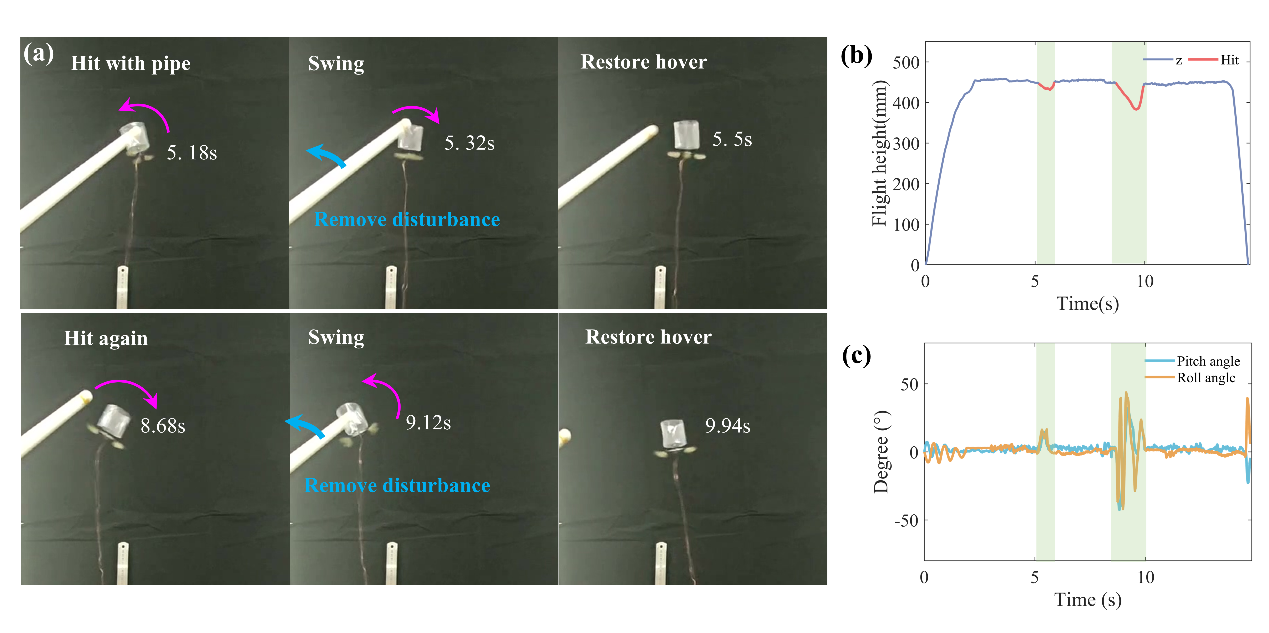


**Supplementary FigureS3.** **Hard contact impact collision recovery flight demonstration of the Tumbler FWMAV:** (a)Flight demonstration and tracking of the robot hit by the PVC pipe twice in succession; (b) The flight tracking height-time fitting curve; (c) The corresponding change in pitch angle and roll angle of the robot; the robot resumes its flight attitude within 1.5s after hard contact collision interference.

**Notes. S4. Lateral Motion Capability Demonstration**

As illustrated in **Supplementary FigureS4. (a)**, by applying distinct driving signals to the two actuators of the FWMAV, a rolling moment is generated around the vertical axis of the actuator's surface. This results in a lateral thrust component that causes the vehicle to exhibit lateral motion, as shown in **Supplementary FigureS4. (b)**. Since the FWMAV does not employ rolling torque control, it will experience self-rotation during lateral movement. (***Supplementary Movies S9***)


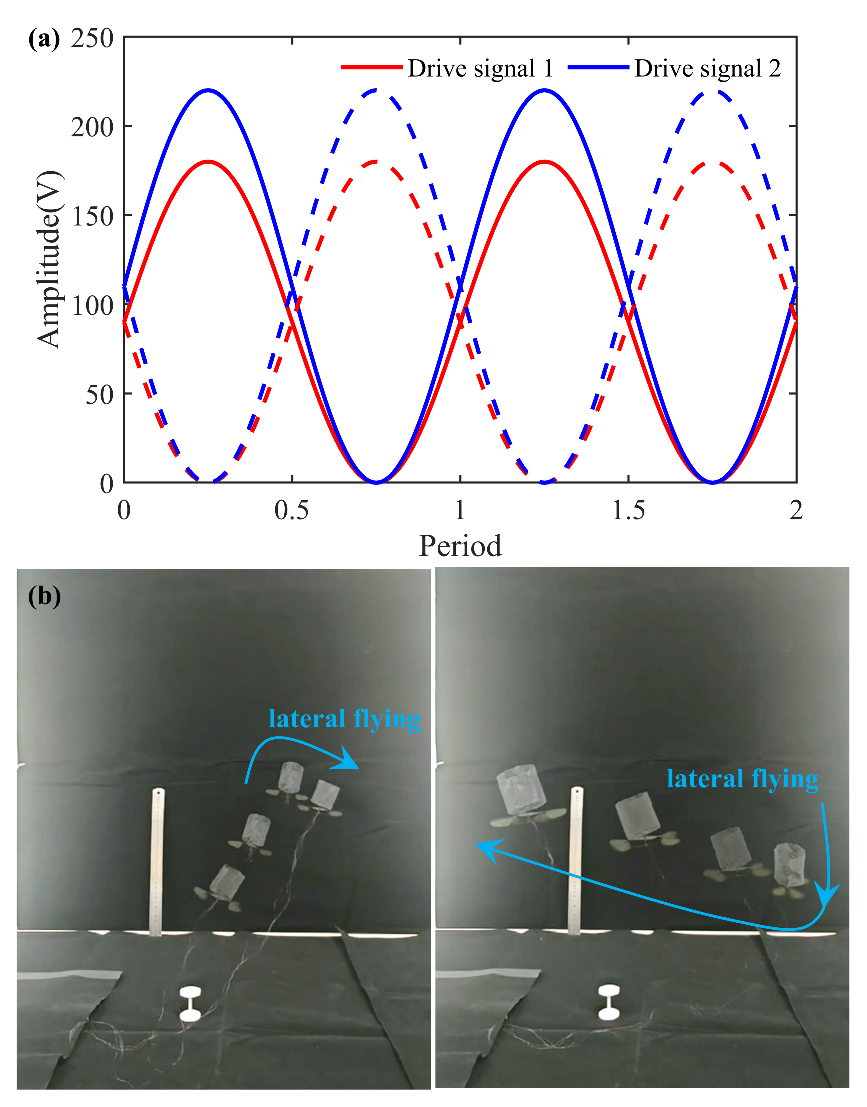


**Supplementary FigureS4.** Demonstration of Lateral Motion of the FWMAV: (a) Control of the driving signals to the two actuators with different amplitudes; (b) Preliminary open-loop control results, demonstrating the FWMAV's effective lateral motion performance.

**Notes. S5. Verification of Yaw Moment Realization**

As illustrated in **Supplementary FigureS5. (a)**, by controlling the high-pressure duty cycle of the two actuators, time asymmetry is introduced into the wing flapping cycle. This asymmetry causes an imbalance in the thrust and damping forces generated by the two wing strokes during flapping, resulting in forces parallel to the flapping direction. These forces produce yaw moments that suppress the robot’s yaw rotation, as shown in **Supplementary FigureS5. (b)**. (***Supplementary Movies S10***)


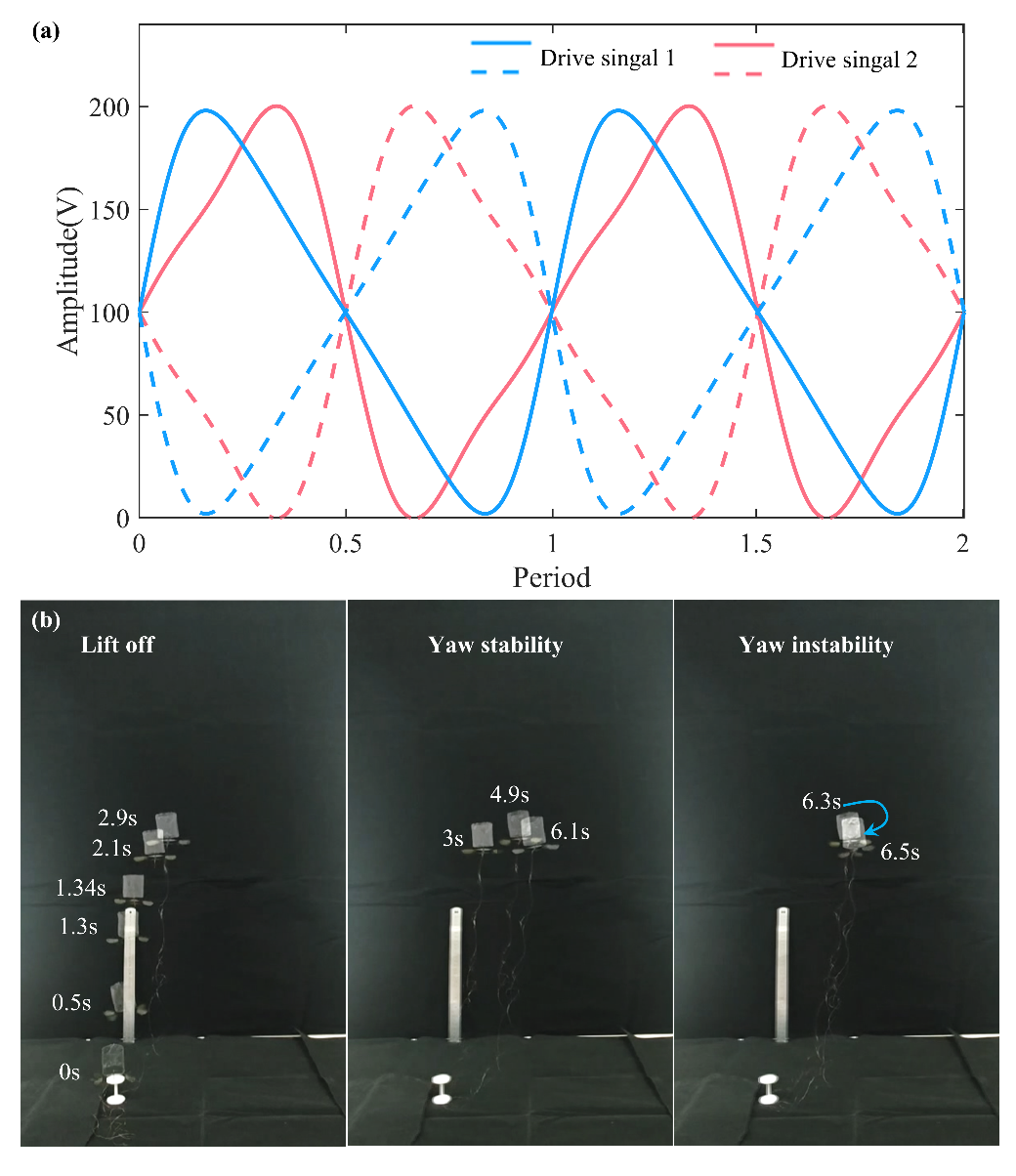


**Supplementary FigureS5.** Introduce control signals to achieve yaw moment: (a) Control the high-pressure duty cycle of the two actuators drive singals; (b) Preliminary open-loop control effect, which can achieve yaw stability for several seconds.

The real-time yaw moment control is not yet implemented, so the FWMAV still experiences a slight deflection after several seconds of stable flight. To address this, we will use a 9-axis IMU to achieve real-time yaw moment control. The 9-axis IMU sensor will be integrated into the designed drive-sensing-control circuit board to collect three-axis acceleration, three-axis angular velocity, and three-axis geomagnetic field data during flight. By applying a nine-axis data fusion algorithm based on the extended Kalman filter, stable and accurate attitude angle calculations can be obtained. Furthermore, adaptive PID control will be employed, allowing the heading angle cascade PID control parameters to dynamically adjust in response to system dynamics, thereby facilitating precise control of the robot's yaw angle.

**Notes. S6. The Scalability of the Application and Environmental Adaptability**

When the robot is tasked with long-duration exploration and monitoring in confined spaces, there may be no designated habitat for it to rest. In such cases, external interference may cause damage to the cylindrical damper, as illustrated in **Supplementary FigureS6. (b)**. To ensure the robot can remain suspended for extended periods, it leverages its surroundings. Specifically, the robot can hover beneath the ceiling by isolating itself from the ceiling via the cylindrical damper. This design prevents disturbance to the robot’s flapping wings, enabling continuous operation. As demonstrated in the **Supplementary FigureS6. (a)**, by extending the robot's input signal wire and supplying appropriate driving signals (240 V, 80 Hz, 120 V), the robot is capable of hovering beneath the ceiling for prolonged durations, even if the cylindrical damper is damaged. (***Supplementary Movies S11***)


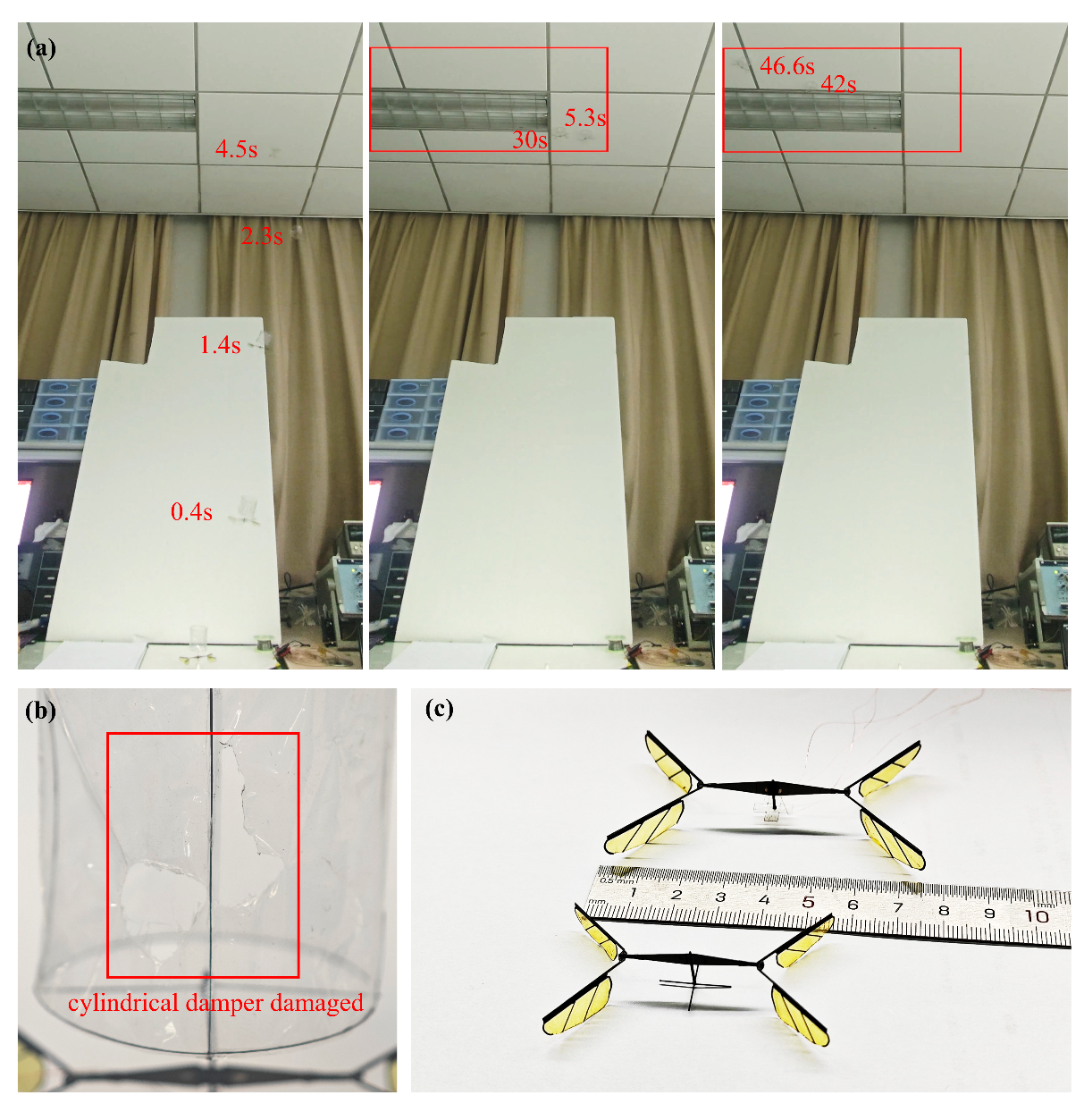


**Supplementary FigureS6.** The scalability and environmental adaptability of FWMAV: (a) The robot can hover below the room ceiling for a long time when the damping cylinder is damaged; (b) Damping cylinders damaged due to external damage; (c) Through the matching design of the actuators and the wings, two prototypes with wingspans of 55mm and 75mm were developed.

In terms of scalability, we have developed two additional prototypes with varying weights and wingspans through the optimized matching of actuators and wings. The wingspans of the prototypes are 55 mm and 75 mm, respectively, as demonstrated in the **Supplementary FigureS6. (c)**, and both exhibit lift-to-weight ratios greater than 2. By using appropriately sized cylindrical dampers, the prototypes can achieve stable, uncontrolled suspension flight. Future research will focus on tailoring FWMAV designs to specific size requirements through detailed optimization of dimensions and actuator matching.

**Notes. S7. Longevity and Maintenance of FWMAV**

**(1) Longevity test**

We performed drive tests on individual piezoelectric actuators under the operational conditions of the FWMAV (200 V, 80 Hz, 100 V). As shown in **Supplementary FigureS7. (a)**, a laser vibration measurement platform was used to record the output displacement of the actuator. Given the limited measurement points of the laser system, we kept the actuator running continuously and repeated the data collection five times to capture displacement output over a continuous 8000-second operation period. The results, as illustrated in **Supplementary FigureS7. (b)**, indicate that the actuator maintains stable output performance after 2.5 hours of continuous operation. Further tests reveal that the actuator can operate continuously for over 24 hours with minimal performance degradation, as illustrated in **Supplementary FigureS7. (c)-(d),** and the operating temperature rises by only 4°C above ambient conditions, as illustrated in **Supplementary FigureS7. (e)**.

To assess the longevity of the FWMAV system itself, we suspended the aircraft using a cable and applied the working conditions (200 V, 80 Hz, 100 V). After overcoming its weight and the tension of the cable, the FWMAV was able to maintain flight at a certain altitude, as illustrated in **Supplementary FigureS7. (f)**. Testing showed that the FWMAV can remain airborne for more than 40 minutes without damage.

**(2) Factors influencing longevity and maintenance methods**

The longevity of the FWMAV is mainly influenced by the output performance and durability of the actuator, as well as the structural integrity of the wings. The piezoelectric actuator is a composite structure. The stability of this composite, the reliability of the connections between layers, and the material properties of the piezoelectric ceramic sheets determine its longevity. The longevity of the wing structure, on the other hand, is predominantly affected by the longevity of the wing membrane and the passive hinge components.

The initial performance of the actuator is mainly influenced by its materials and processing methods. Ensuring optimal initial performance is critical, which is addressed in detail in the process and methodology sections of the manuscript. However, the long-term service longevity of the actuator is also influenced by usage conditions. Operating under high-frequency, high-pressure, high-temperature, high-humidity, or high-pH environments, or under heavy load conditions, will reduce the actuator's actual longevity.


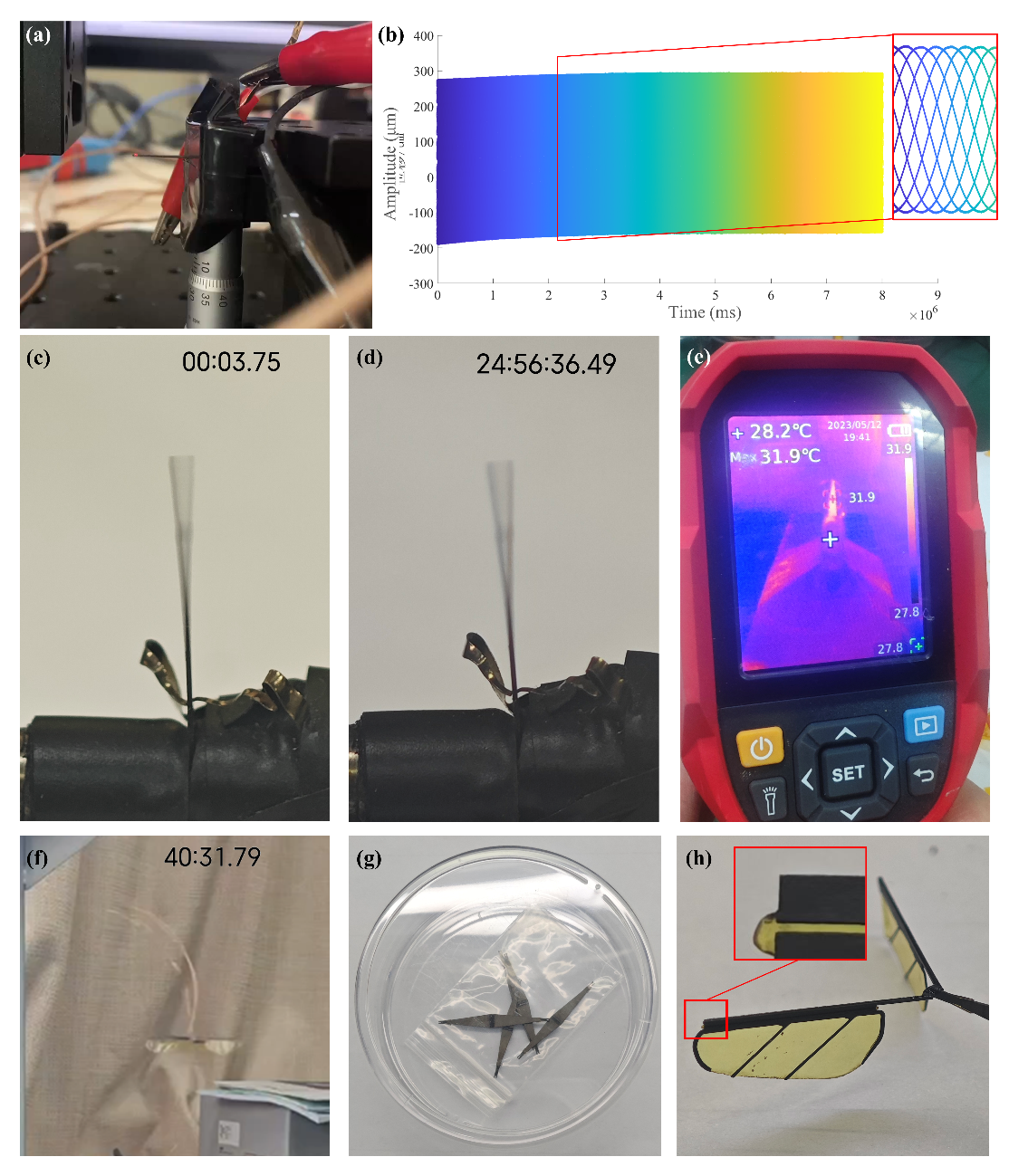


**Supplementary FigureS7.** Service longevity test and maintenance of FWMAV: (a) Actuator output characteristics were assessed using laser vibration measurement; (b) Amplitude characteristics of the actuator output over 8,000 seconds; (c)-(d) Idle actuator output at driving conditions of 200 V, 80 Hz, and 100 V, showing negligible attenuation after continuous operation for over 24 hours; (e) The actuator temperature increased by approximately 4°C after continuous operation for 24 hours; (f) Under driving conditions of 200 V, 80 Hz, and 100 V, the FWMAV is capable of sustained flight for over 40 minutes per single cycle without damage; (g) Unused actuators should be stored in a sealed bag at room temperature; (h) A circular transition structure is designed at the stress concentration zones during wing movement to enhance the service life of the wings.

The voltage tolerance of the actuator designed for this system exceeds 300 V, with the optimal operating frequency for the actuator when loaded with wings being below 100 Hz. To maximize the service longevity of the actuator, it is recommended that the driving voltage be kept below 280V and the operating frequency below 100 Hz. Additionally, the actuator should be used in a dry environment, and unused actuators should be stored in a sealed bag at room temperature, as illustrated in as illustrated in **Supplementary FigureS7. (g)**.

The longevity of the wing structure is chiefly influenced by the processing techniques used and external factors such as interference during operation. While the films used in the wing structure possess excellent mechanical properties, stress concentrations and processing burrs can easily form at the junctions between the wing membrane and the wing veins. During high-frequency oscillations, the wing membrane is susceptible to tearing, which can negatively impact the FWMAV's longevity. To mitigate these risks, we have designed a circular over structure at the stress-concentration areas and applied adhesive at the burrs, as shown in as illustrated in **Supplementary FigureS7. (h)**. This approach enhances the structural strength of the wings, thereby ensuring their durability.

Finally, since the overall structural strength of the robot is relatively low, it is crucial to avoid external force-induced squeezing or tearing during storage and use, as such actions may cause damage to the FWMAV's structure or components, potentially reducing its service longevity.
